# The chromosome-level genome of *Mesobuthus martensii* provides insights into its evolution and the diversity of venom peptides

**DOI:** 10.1016/j.pscia.2026.100149

**Published:** 2026-09-06

**Authors:** Daixi Liu, Qianhui Zeng, Yifei Sun, Changfen Li, Yanjun Song, Zhuo Shang, Kunlong Li, Hongjie Zhu, Feng Li, Chuanbin Shen, Jianhua Ju

**Affiliations:** a State Key Laboratory of Discovery and Utilization of Functional Components in Traditional Chinese Medicine, Key Laboratory of Chemical Biology (Ministry of Education), Shandong Basic Science Research Center (Pharmacy), School of Pharmaceutical Sciences, Cheeloo College of Medicine, Shandong University, Jinan, 250012, China; b Key Laboratory of Marine Drugs, Chinese Ministry of Education, School of Medicine and Pharmacy, Ocean University of China, Qingdao, 266000, China

**Keywords:** *Mesobuthus martensii*, *Quanxie*, Chromosome-level genome, Venom peptide, Antithrombotic peptide

## Abstract

*Mesobuthus martensii*, the source species of the traditional Chinese medicine “Quanxie”, has long been utilized for its therapeutic properties. Venom peptides are recognized as the major active molecular basis of these pharmacological activities. Despite this, their development as therapeutic agents remains poorly explored. In this study, we performed an integrated multi-omics investigation to systematically explore the venom peptides of *Mesobuthus martensii*. We generated a chromosome-level genome assembly of *Mesobuthus martensii* using third-generation sequencing technologies, yielding a genome size of 1.08 Gb with a contig N50 of 46.46 Mb. Integrated genomic and transcriptomic analyses led to the identification of 51 putative novel venom peptide candidates. From the broader venom peptide set, five lysine- and arginine-rich candidates were selected for recombinant expression and functional characterization. Functional evaluation revealed that MmTX67, MmTX106 and MmTX126 modulated coagulation-related parameters *in vitro*, with MmTX106 further demonstrating the most pronounced antithrombotic effect *in vivo*. Collectively, these findings provide novel insights into the genetic basis of bioactive substances in “Quanxie” and highlight MmTX106 as the most promising antithrombotic candidate, while providing a molecular foundation for further evaluation of other scorpion venom-derived peptide candidates.

## Graphical abstract

A high-quality, chromosome-level genome of *Mesobuthus martensii* was assembled, leading to the identification of 51 putative novel venom peptide candidates. Five Lys/Arg-rich venom peptides were then expressed and functionally evaluated. MmTX67, MmTX106, and MmTX126 modulated coagulation-related parameters *in vitro*, while MmTX106 showed the most pronounced and statistically significant antithrombotic effect *in vivo*.

**Figure:**
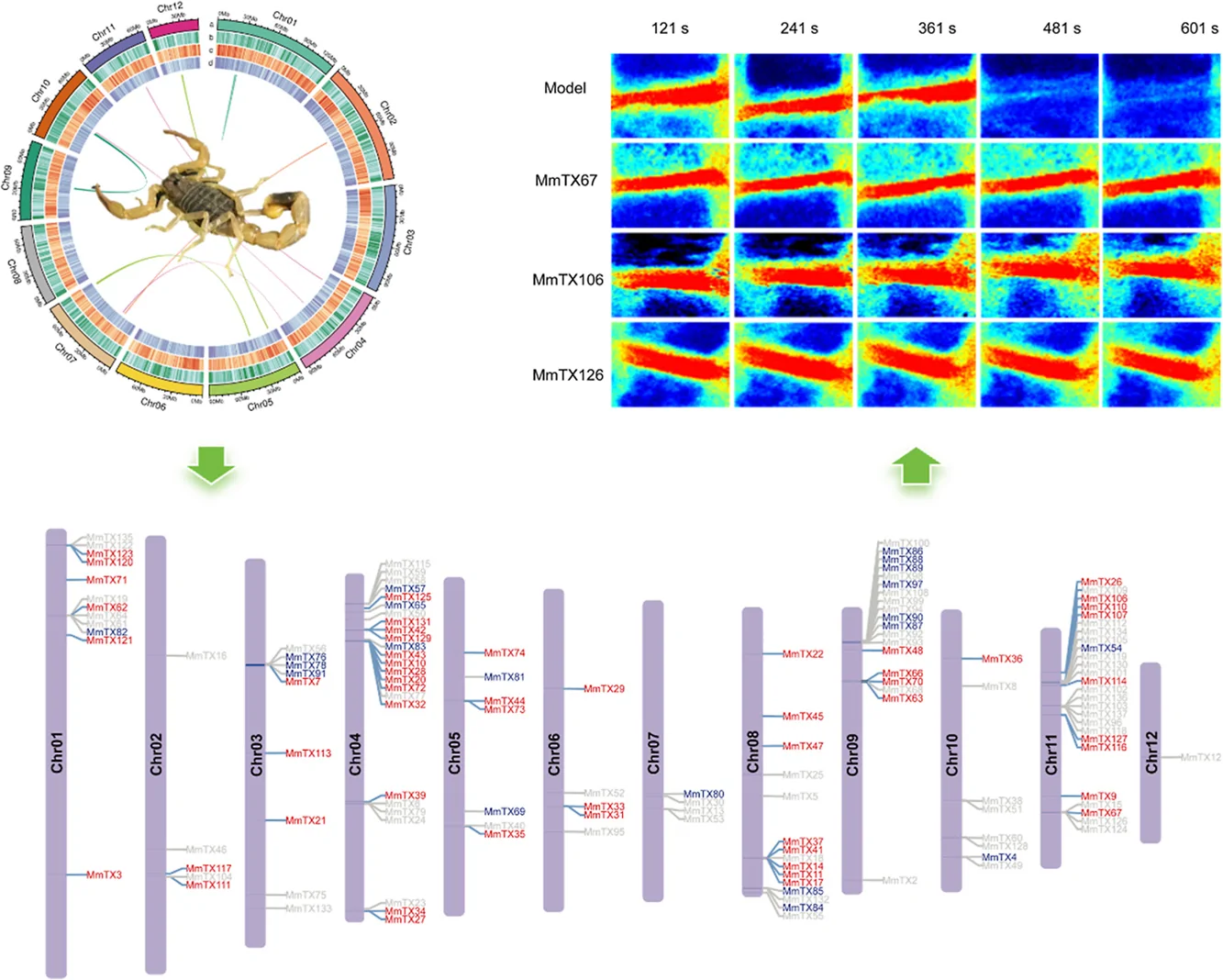

## 1 Introduction

*Mesobuthus martensii*, a representative species of the order Scorpiones (Arthropoda), is widely distributed across East Asia and serves as a crucially important model organism in scorpion research [1]. As one of the oldest terrestrial arthropods, with an evolutionary history dating back over 400 million years, scorpions hold immense ecological and evolutionary significance. *M. martensii* plays a crucial role as a predator in assorted ecosystems and occupies a unique position in toxicological and medical studies. In traditional Chinese medicine (TCM), the dried body of *M. martensii* (referred to as “Quanxie”) has been historically utilized for its purported effects of "dispelling wind, relieving spasms, dredging collaterals, alleviating pain, and detoxifying." It is commonly prescribed for conditions such as convulsions, pediatric infantile convulsions, stroke, rheumatic arthritis, and inflammatory lesions. Modern pharmacological research has identified a wide range of bioactive components in “Quanxie” [2], including large bioactive peptides like venom peptides and small molecules such as amino acids, phospholipids, fatty acids, sterols, and alkaloids [[3], [4], [5], [6], [7]]. Despite these advances, the full spectrum of pharmacologically active molecules in *M. martensii* remains to be fully characterized. Notably, venom peptides which are the primary bioactive constituents responsible for the therapeutic properties of this medicinal arthropod remain largely underexplored.

Scorpion venoms harbor a diverse array of bioactive peptides. These are classified into disulfide-bridged peptides (DBPs), which target voltage-gated ion channels (Nav, Kv, Cav, Clv), and non-disulfide-bridged peptides (NDBPs), which exert antimicrobial, antiviral, and anticancer effects via membrane disruption [6,8]. Analgesic peptides have been extensively studied, with BmK AS, BmK IT2, and BmK AGAP modulating Nav1.7, Nav1.8, and Nav1.9 channels to exhibit potent pain relief comparable to morphine without addiction [9,10]. In contrast, antithrombotic peptides remain underexplored, although recent discoveries such as Leu-Trp/Ile-Trp from *Heterometrus laoticus* and TanP from *Tityus stigmurus* have demonstrated anticoagulant effects via platelet inhibition or thrombin modulation [11,12]. Despite these advances, no scorpion venom peptide-derived drug has yet reached the market. Currently, only chlorotoxin, an active peptide derived from *Leiurus quinquestriatus*, has been developed as a tumor probe and advanced to clinical trials, owing to its specific binding affinity for glioma cells [13]. Given the traditional use of “Quanxie” for blood stasis-related disorders, systematic investigation of antithrombotic peptides is urgently needed to fully realize the therapeutic potential of this valuable natural resource.

Traditional discovery of venom peptides relies on isolation and purification, an approach characterized by low efficiency. The advent of modern omics technologies, especially transcriptomics and proteomics [14,15], has dramatically increased the rate of discovery of venom peptides. However, genome mining remains limited by the lack of chromosome-level genomic data in *M. martensii*, which especially hinders the systematic analysis of novel venom peptides [16,17]. Genomic sequencing enables systematic elucidation of the genetic blueprint underlying pharmacologically active compounds in medicinal animals, providing mechanistic insights critical for drug discovery [[18], [19], [20]]. Although chromosome-level genome assemblies have recently been reported for several scorpion species, including *Hadrurus arizonensis*, *Mesobuthus przewalskii*, and *Belisarius xambeui* [[21], [22], [23]], a chromosome-level genome assembly of *M. martensii* has not yet been available. The genome of *M. martensii* was first sequenced in 2013, but it was assembled only at the contig level, with an N50 of 45.2 Kb [24]. Other scorpion genomic resources, such as those of *Centruroides vittatus*, *Centruroides sculpturatus*, and *Androctonus mauritanicus*, are also limited by scaffold/contig-level continuity or incomplete annotations [25,26]. These limitations have hindered in-depth analyses of genome architecture, gene family evolution, regulatory networks, and venom peptide diversity in *M. martensii*. Third-generation sequencing (TGS) technologies, such as PacBio and Oxford Nanopore sequencing, together with Hi-C or related chromosome-conformation approaches, provide effective solutions for assembling complex genomes, particularly those rich in repetitive sequences [27]. Therefore, a high-quality chromosome-level genome assembly of *M. martensii* is still needed to support systematic analyses of genome organization, venom peptide diversity, and the genetic basis of pharmacologically active compounds in this medicinal scorpion.

In this study, to elucidate the genetic architecture underlying scorpion-derived pharmacologically active compounds, we employed TGS to generate a chromosome-level genome assembly of *M. martensii*. Furthermore, we identified 51 putative novel venom peptide candidates and verified the anticoagulant activity of selected candidates. In summary, our results provide a framework for understanding arthropod evolutionary adaptations and establish a resource for the discovery and further optimization of scorpion venom-derived antithrombotic peptide candidates.

## 2 Materials and methods

### 2.1 Genome sequencing, assembly, and annotation

Live specimens of *M. martensii* were collected from the Weifang region of Shandong province. Muscle tissue samples were obtained and rapidly frozen in liquid nitrogen to preserve their original state. High-quality genomic DNA was extracted, and whole genome sequencing was conducted with PacBio high-throughput sequencing technology. Hi-C technology aided in anchoring contigs by generating clean read pairs mapped to a polished genome, facilitating scaffolding and filtering invalid reads. Contig clustering and ordering were achieved using Lachesis, resulting in a high-quality assembly with specified chromosomal lengths. Transposable elements and tandem repeats were annotated through homology-based and de novo methods, while protein-coding genes were predicted using a combination of approaches. Functional annotation was based on alignments to various databases, and non-coding RNA genes were identified using specific algorithms. Pseudogenes were predicted after masking functional genes, analyzing for stop and frame-shift mutations.

### 2.2 Genome evolution analysis

The protein-coding sequences in the *M. martensii* genome and genomes from other species were compared using OrthoFinder (v2.4.0). The Pfam V33.1 database was used to annotate the obtained gene families. To reveal phylogenetic relationships among *M. martensii* and other closely related species, protein sequences of single-copy ortholog gene from *M. martensii* were used for phylogenetic tree reconstruction. The protein sequences of the single-copy ortholog genes were aligned with mafft (v7.205) program, and the corresponding Coding DNA Sequences (CDS) alignments were generated and concatenated with the guidance of protein alignment. IQ-TREE (v2.2.0) was used to construct the phylogenetic tree. The phylogenetic relationship of other closely related species was consistent with previous studies. Based on the identified gene families and the constructed phylogenetic tree with predicted divergence time of those species, we used CAFE (v4.2) to analyze gene family expansion and contraction. In CAFE, a random birth and death model is proposed to study gene gain or loss in gene families across a specified phylogenetic tree. Then, conditional *P*-value was calculated for each gene family, and family with conditional *P*-value less than 0.05 was considered to have an accelerated rate for gene gain or loss. These expansion and contraction gene families (*P*-value ≤ 0.05) were mapped to KEGG pathways for functional enrichment analysis, which was conducted using the enrichment methods. This method implemented hypergeometric test algorithms and the *Q*-value (FDR, False Discovery Rate) was calculated to adjust the *P*-value using R package (https://github.com/StoreyLab/qvalue).

### 2.3 RNA extraction, quantification and qualification

The total RNA was extracted according to the instruction manual of the TRIzol Reagent (Life technologies, California, USA). RNA concentration and purity were measured using NanoDrop 2000 (Thermo Fisher Scientific, Wilmington, DE). RNA integrity was assessed using the RNA Nano 6000 Assay Kit of the Agilent Bioanalyzer 2100 system (Agilent Technologies, CA, USA).

### 2.4 Library preparation for transcriptome sequencing

A total amount of 1 μg RNA per sample was used as input material for the RNA sample preparations. Sequencing libraries were generated using Hieff NGS Ultima Dual-mode mRNA Library Prep Kit for Illumina (Yeasen Biotechnology (Shanghai) Co., Ltd.) following manufacturer's recommendations and index codes were added to attribute sequences to each sample. The libraries were sequenced on an Illumina NovaSeq platform to generate 150 bp paired-end reads, according to the manufacturer's instructions.

### 2.5 Transcriptome data analysis

The raw reads were further processed with a bioinformatic pipeline tool, BMKCloud (www.biocloud.net) online platform. Clean data (clean reads) were obtained by removing reads containing adapter, reads containing poly-N and low-quality reads from raw data. These clean reads were then mapped to the reference genome sequence. HISAT 2 software was used to map with reference genome. The StringTie Reference Annotation Based Transcript (RABT) assembly method was used to construct and identify both known and novel transcripts from HISAT 2 alignment results Gene function was annotated based on the following databases: Nr (NCBI non-redundant protein sequences); Pfam (Protein family); KOG/COG (Clusters of Orthologous Groups of proteins); Swiss-Prot (A manually annotated and reviewed protein sequence database); KO (KEGG Ortholog database); GO (Gene Ontology). Gene expression levels were estimated by fragments per kilobase of transcript per million fragments mapped.

### 2.6 Mining and functional validation of venom peptides

Venom peptides were identified using HMMER v3.0.0 and BLASTP (E-value cutoff < 1e-5). Query sequences were retrieved from UniProt based on published datasets. The chromosomal locations of the identified venom peptides were visualized using TBtools [28,29], and their expression level heatmaps were generated using the Chiplot online platform (https://www.chiplot.online). To validate the biological activities of these venom peptides, five candidate peptides were selected for recombinant expression and functional characterization using an integrated prioritization strategy. Enrichment in lysine and arginine residues was used as the primary criterion, because Lys/Arg-rich sequences may favor electrostatic interactions with negatively charged catalytic pockets or membrane-associated coagulation reaction surfaces, both of which are relevant to coagulation regulation. On this basis, we further considered transcript abundance, sequence novelty, predicted peptide family, cysteine framework, sequence length, and feasibility of recombinant expression. The five candidates were selected to represent a balanced distribution of these features as far as possible, rather than being chosen solely according to Lys/Arg content. The open reading frames (ORFs) of MmTX67, MmTX70, MmTX106, MmTX118, and MmTX126 were PCR-amplified from *M. martensii* cDNA and cloned into the multiple cloning site (MCS) of the pET-32a vector. Recombinant plasmids were transformed into *E. coli* SHuffle T7 competent cells. Positive clones were confirmed by PCR and Sanger sequencing. The primer sequences used are listed in Table S4.

For protein expression, seed cultures were prepared by inoculating single colonies into 5 mL LB medium with antibiotic at 37 °C for 16–18 h. A 1 mL aliquot was transferred to 100 mL LB, cultured to logarithmic phase (OD600 = 0.6–0.8), then scaled up to 1000 mL. Expression was induced with IPTG at 16 °C for 18–20 h. Cells were harvested by centrifugation, and pellets stored at −80 °C. For purification, cells were lysed, centrifuged, and the supernatant loaded onto a Ni-NTA column. After elution, proteins were desalted, digested with enterokinase to remove the TrxA tag, and further purified by sequential Ni-NTA, FPLC, and HPLC. Purity was confirmed by Tricine-SDS-PAGE.

### 2.7 Antithrombotic activity assay

The *in vitro* anticoagulant activities of scorpion venom peptides MmTX67, MmTX70, MmTX106, MmTX118 and MmTX126 were evaluated using thromboelastography (TEG), recalcification assay, activated partial thromboplastin time (APTT), prothrombin time (PT), and thrombin time (TT) tests. For TEG assay, 32 μL reconstituted peptides were incubated with 310 μL platelet-poor plasma (PPP) at 37 °C for 5 min. Subsequently, 20 μL CaCl_2_ (450 μmol/L) was added to the mixture, and clot formation was monitored using a thromboelastograph. For the recalcification assay, peptide solutions were pre-mixed with 50 μL PPP and incubated at 37 °C for 10 min. Clotting was initiated by adding 30 μL CaCl_2_-Hepes buffer, and absorbance was measured at 405 nm using a microplate reader. For APTT/PT/TT tests, peptide-treated PPP (50 μL final volume) was analyzed using an automated coagulation analyzer. For APTT, 50 μL peptide-PPP mixture was incubated with 50 μL APTT reagent at 37 °C for 3 min, followed by 50 μL CaCl_2_ (25 mmol/L). PT and TT were determined by adding 50 μL thromboplastin reagent or thrombin solution (100 NIH U/mL), respectively. Negative controls included solvent-only treatments (PBS or DMSO), while positive controls used enoxaparin sodium (25–50 μmol/L). All experiments were performed in triplicate. Data were analyzed using manufacturer software, with clotting parameters compared against controls. For assays involving more than two groups, statistical significance was determined using one-way ANOVA, and *P* < 0.05 was considered statistically significant.

*In vivo* experiment, a ferric chloride (FeCl_3_)-induced carotid artery thrombosis model in mice was established. The antithrombotic effect was assessed by monitoring carotid blood flow, measuring the time to complete vascular occlusion, and calculating the area under the blood flow curve. Mice were anesthetized with 2% isoflurane, placed in a supine position, and secured to a platform. The fur on the neck was shaved, and a midline incision of approximately 1 cm was made using scissors. The left common carotid artery was carefully isolated using blunt forceps. A piece of filter paper (6.0 mm × 2.0 mm) was placed under the isolated artery to separate it from the underlying tissue. The test peptide was administered via tail vein injection. MmTX67, MmTX106, and MmTX126 were administered at 15, 10, and 15 mg/kg, respectively. Immediately after administration, a FeCl_3_-soaked suture (2 mm in length) was applied topically to the adventitial surface of the left common carotid artery for 2 min and then removed. Carotid blood flow was monitored using a RWD laser speckle. The time to first complete vascular occlusion was recorded. If occlusion did not occur during the observation period, the data were recorded based on the total monitoring time. The blood flow data obtained were analyzed to determine the occlusion status of the carotid artery in each treatment group. The study was conducted with 5 male ICR mice per group, with body weights of approximately 20 g. Animal experiments were designed and reported in accordance with the ARRIVE guidelines. Mice were purchased from Shandong Pengyue Experimental Animal Technology Co., Ltd (Jinan, China). Animal were maintained under specific pathogen-free conditions with controlled temperature (20 ± 2°C) with humidity (relative humidity at 55–60%), and 12 h light/dark cycles and free access to food and water. All animal experiments were approved by the Institutional Animal Care and Use Committee of Shandong University (approval No. YXDW2025-0099) and were conducted in accordance with institutional animal care guidelines. Mice were randomly allocated to each experimental group, and outcome assessment was performed in a blinded manner where feasible. Efforts were made to minimize animal suffering and animal use.

### 2.8 Statistical analysis

Data are presented as mean ± standard deviation (SD) unless otherwise indicated. Statistical analyses were performed using GraphPad Prism 10.4.0. For comparisons involving more than two groups, one-way analysis of variance (ANOVA) was used, followed by appropriate multiple-comparison analysis when applicable. A value of *P* < 0.05 was considered statistically significant. The number of biological replicates or animals is indicated in the corresponding figure legends.

## 3 Results

### 3.1 Sequencing, assembly and annotation of *M. martensii* genome

We deployed third-generation sequencing technology to achieve a high-contiguity genome assembly of *M. martensii*. Genomic DNA was extracted from an adult male *M*. *martensii* captured from the wild in Weifang City, Shandong Province, China. Second-generation and third-generation sequencing were adopted for the comprehensive analysis of the *M. martensii* genome. A PacBio library was constructed using the genomic DNA and sequenced to obtain approximately 50.49 Gb of clean data. The total sequencing depth was approximately 45 X, with a read N50 of 14.26 Kb and an average read length of 10.52 Kb. Hifiasm software (version 0.16) was then used for assembly. The total length of the genomic Contig sequence was 1.08 Gb using Hifiasm splicing, and the Contig N50 was 46.46 Mb. Three methods were applied in order to evaluate the genome assembly. Firstly, the metazoa database of OrthoDB 10 was selected and BUSCO assessed completeness at 97.0%. Secondly, the second generation read back ratio was 99.29%, coverage was 99.98%, and average sequencing depth was 113. Thirdly, the third-generation reads back ratio was 99.65%, the coverage was 99.99%, and the average sequencing depth was 47. Taken together, these data suggested a high level of integrity and accuracy for the constructed assembly.

Continuing further, a Hi-C library was constructed for chromosomal-level genome assembly, and 140.17-Gb filtered clean reads were used for the following analysis. As a result, a total of about 1067.96 Mb length of genome bases were successfully anchored to 12 chromosomes, accounting for 99.26% (Fig. 1A). After Hi-C error correction, auxiliary chromosome mounting, and redundancy removal, the final version of the genome was obtained, and the assembly results were 1.1 Gb for the reference genome size, 46.46 Mb for Contig N50, and 86.33 Mb for Scaffold N50. Similarly, the Hi-C assembly results were also evaluated, and the results showed that the interaction intensity between neighboring sequences (diagonal positions) in the chromosome results of Hi-C assembly was high, whereas the interaction signal intensity between non-neighboring sequences (non-diagonal positions) was weak, consistent with the principle of Hi-C assisted genome assembly (Fig. 1B).

**Fig. 1:**
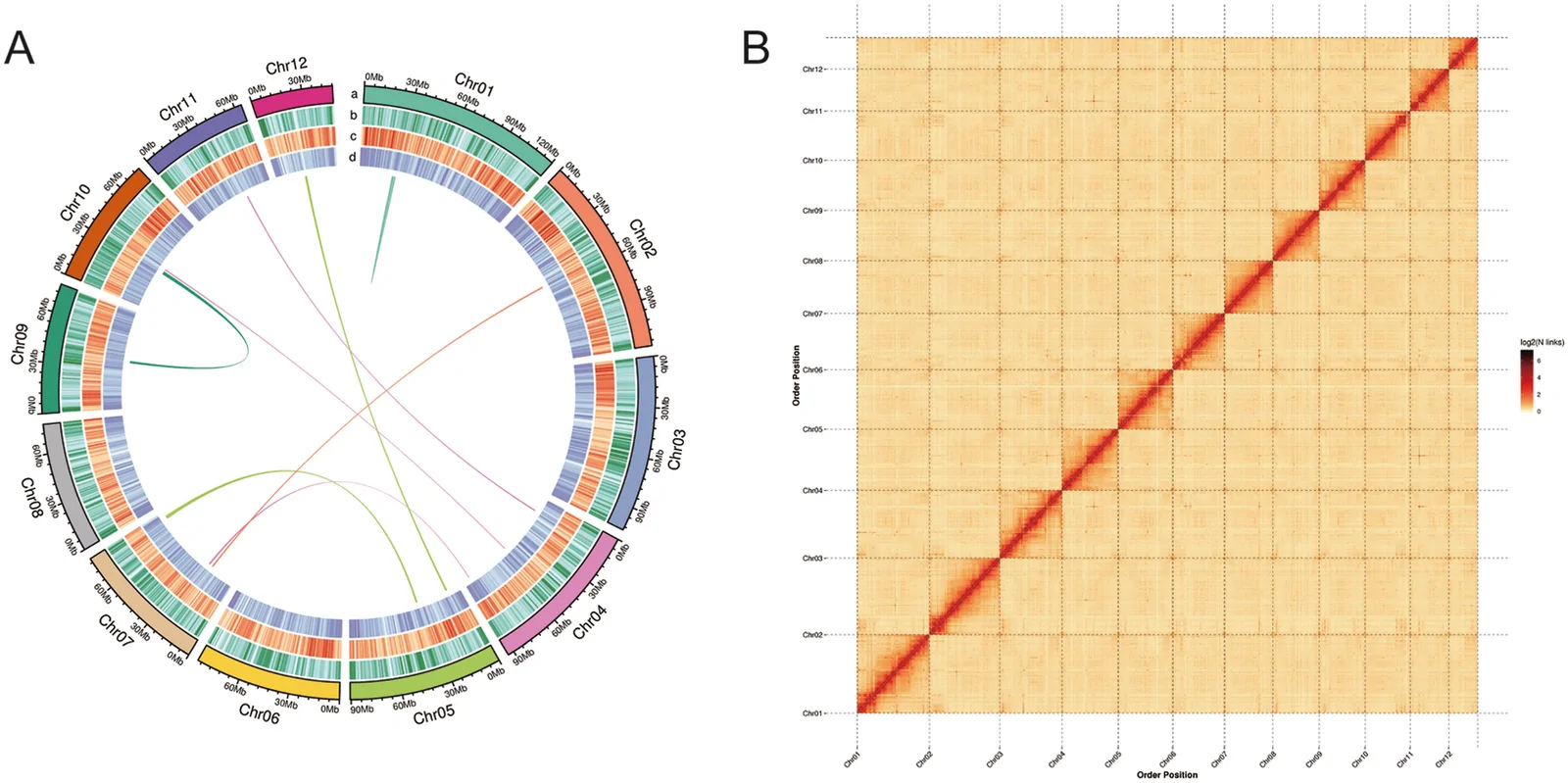
Genome assembly and comparative analysis of *M. martensii*. (A) Circos plot of distribution of the genomic elements in *M. martensii*. (B) Heatmap of chromosome interactions in *M. martensii*.

The genome of *M. martensii* at the contig level had been reported in 2013. Here, we compared our version with the old version by genomic synteny analysis (Table S1). At an overall level, 96% of our version matched up to the 2013 version. The contig N50 of the 2013 version was 45.2 Kb, whereas in the 2026 version it was 46.46 Mb. The percentage of BUSCO in the 2013 version was only 57.13%, whereas in the 2026 version it was 97%. In summary, the genome of *M. martensii* has been largely improved by virtue of the recent discovery efforts.

Genome annotation was performed using a combination of three gene prediction methods, specifically homology prediction, de novo prediction and transcriptome prediction. For transposable elements (TE), about 456,836,858 bp were ultimately obtained, accounting for 42.46% of the assembled genome. For non-coding RNA, 14,422 tRNAs, 454 rRNAs, and 12 miRNAs were obtained by prediction. For pseudogene analysis, 474 pseudogenes were obtained. For protein-coding genes, the *M. martensii* genome was predicted to contain 26,533 protein-coding genes, of which 87.19% could be annotated using NR, GO, KEGG and other known protein databases. The genetic characteristics of predicted protein-coding genes was compared with the closely related species. The subsequent results indicated that the gene length, exon length and number, intron length and number, and coding region (CDS) length showed high similarity. Meanwhile, the BUSCO (v5.2.2) software was used to assess the completeness of gene predictions, where 96.44% of the BUSCO genes were present in predicted genes. In conclusion, the above results indicated that the predicted genes were of high integrity and good quality.

### 3.2 Comparative genomics and evolution

Gene family clustering analysis showed that the number of gene families shared by *M. martensii* and *Centruroides sculpturatus* (*C. sculpturatu*s) was 9140 (78%), with *Hylyphantes graminicola* (*H. graminicola*) this number was 5972 (51%), with *Apis cerana* (*A. cerana*) it was 4258 (36%), and with *Caenorhabditis elegans* (*C. elegans*) it was 2421 (21%) (Fig. 2A). 1407 common gene families were found in these species, and 1341 gene families were unique to *M. martensii*. The species-specific gene families were subjected to GO and KEGG enrichment analysis using clusterProfiler v3.4.4 (Fig. 2B&C). The results of the GO enrichment analysis revealed that the species-specific gene families were primarily enriched in processes such as transposase activity, RNA-directed DNA polymerase activity, RNA-DNA hybrid ribonuclease activity, toxin activity, fatty acid synthase, and elongase activity (Fig. 2B). Similarly, the KEGG enrichment analysis showed that these gene families were predominantly enriched in pathways such as those associated with ABC transporters, lysine degradation, fatty acid elongation, and biosynthesis of unsaturated fatty acids (Fig. 2C).

**Fig. 2:**
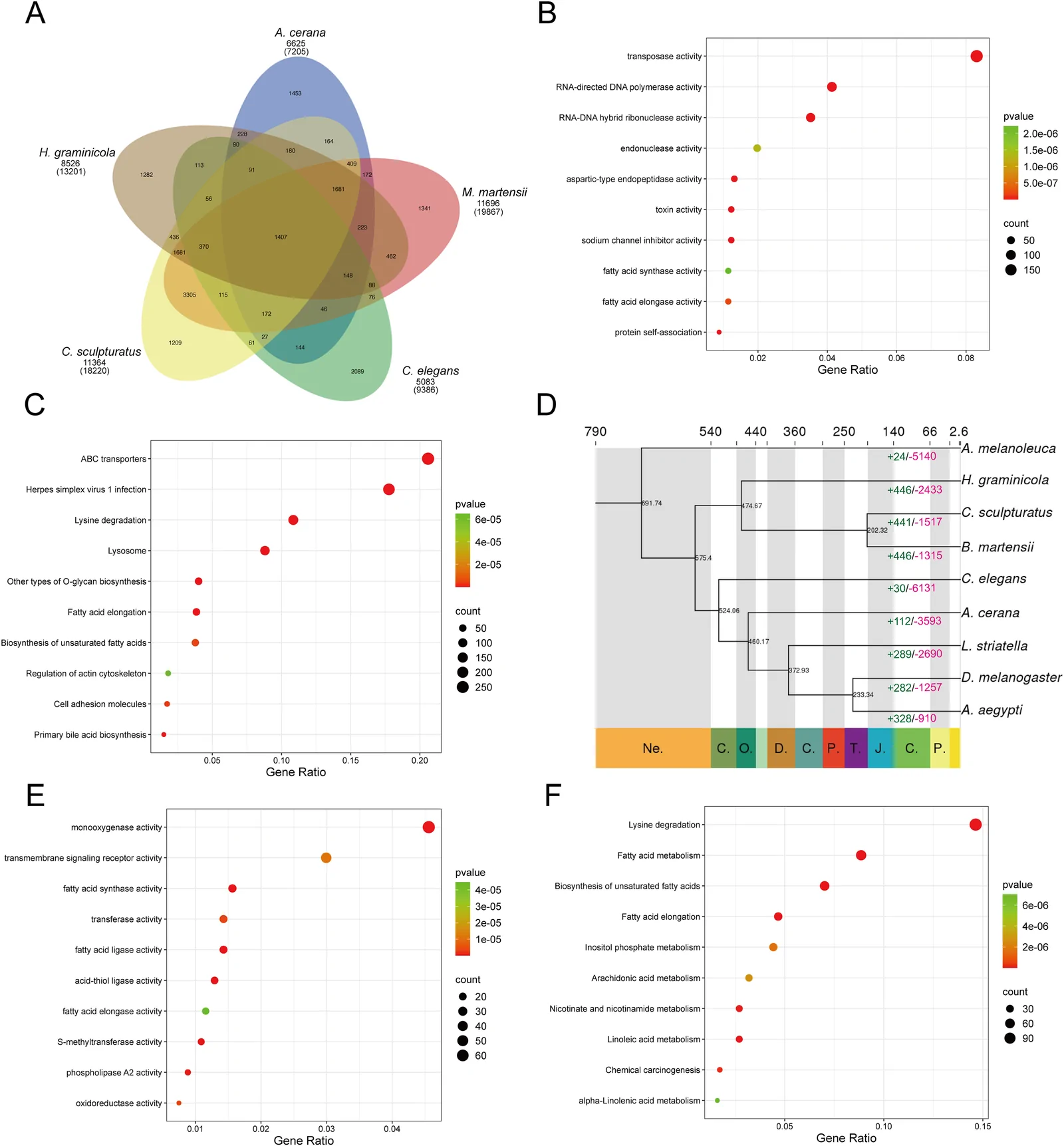
Comparative Genomics of *M. martensii*. (A) Venn diagram of gene family clustering. The number below the species name is the total number of gene families, with the corresponding number of genes in parentheses; the number in the Venn diagram is the number of gene families. (B) GO clustering analysis of the unique gene family. (C) KEGG clustering analysis of the unique gene family. (D) Phylogenetic tree with differentiation time. (E) GO clustering analysis of the expansion gene family. (F) KEGG clustering analysis of the expansion gene family.

Furthermore, a phylogenetic tree was constructed with the obtained 354 single-copy gene families using the maximum likelihood method (Fig. 2D). *Ailuropoda melanoleuca* (*A. melanoleuca*) was selected as the outgroup, and *M. martensii* was on the same branch as *C. sculpturatus*. With the help of fossil time correction, a species tree with divergent times could be obtained. The differentiation time of *M. martensii* and *C. sculpturatus* was about 202 million years ago (Fig. 2D). Using a phylogenetic tree with divergence times and the results from gene family clustering, we estimated the number of ancestral gene family members for each branch using a birth-death rate model. Based on this, we predicted the contraction and expansion of gene families in this species relative to its ancestors (defined as significant if both the family-wide P-values and Viterbi P-values were less than 0.05). The results showed that the number of contracted and expanded gene families in *M. martensii* was 1315 and 446, respectively (Fig. 2D). The GO enrichment analysis indicated that the expanded gene families were primarily enriched in processes such as monooxygenase activity, fatty acid ligase activity, S-methyltransferase activity, acid-thiol ligase activity, and fatty acid synthase activity (Fig. 2E). Similarly, the KEGG enrichment analysis revealed that the expanded gene families were mainly enriched in pathways such as those involved in lysine degradation, biosynthesis of unsaturated fatty acids, fatty acid metabolism, fatty acid elongation, and linoleic acid metabolism (Fig. 2F).

### 3.3 Genetic diversity of venom peptides

A total of 136 venom peptides were identified through a Profile Hidden Markov Model (HMM)-based homology search (Table S3). The venom peptides were ubiquitously distributed across all 12 chromosomes, with significant enrichment observed on chromosome 1 (24 peptides), chromosome 6 (28 peptides), and chromosome 11 (26 peptides) (Fig. 3A). Notably, 31 peptides exhibited 100% sequence identity with previously characterized venom peptides (Blue-labeled entries in Fig. 3A). Applying stringent sequence alignment criteria (Clustal Omega v1.2.4; BLOSUM62 matrix, E-value <1e-5), we classified 51 novel peptides, with <70% amino acid similarity to known venom peptides (Red-labeled entries in Fig. 3A). Transcriptome analysis revealed distinct expression patterns across tissues. Consistent with their biological roles, the majority of peptides showed predominant expression in venom glands, with negligible sexual dimorphism between male and female specimens (Fig. 3B and C). Intriguingly, some peptides were exclusively detected in non-venom tissues, suggesting that these peptides may have functions beyond envenomation. Notably, novel peptides exhibited significantly lower expression levels compared to conserved counterparts (Fig. 3B and C), a phenomenon potentially attributable to traditional methods of venom peptide discovery relying on isolation and purification, with low expression levels limiting the efficiency of their discovery.

**Fig. 3:**
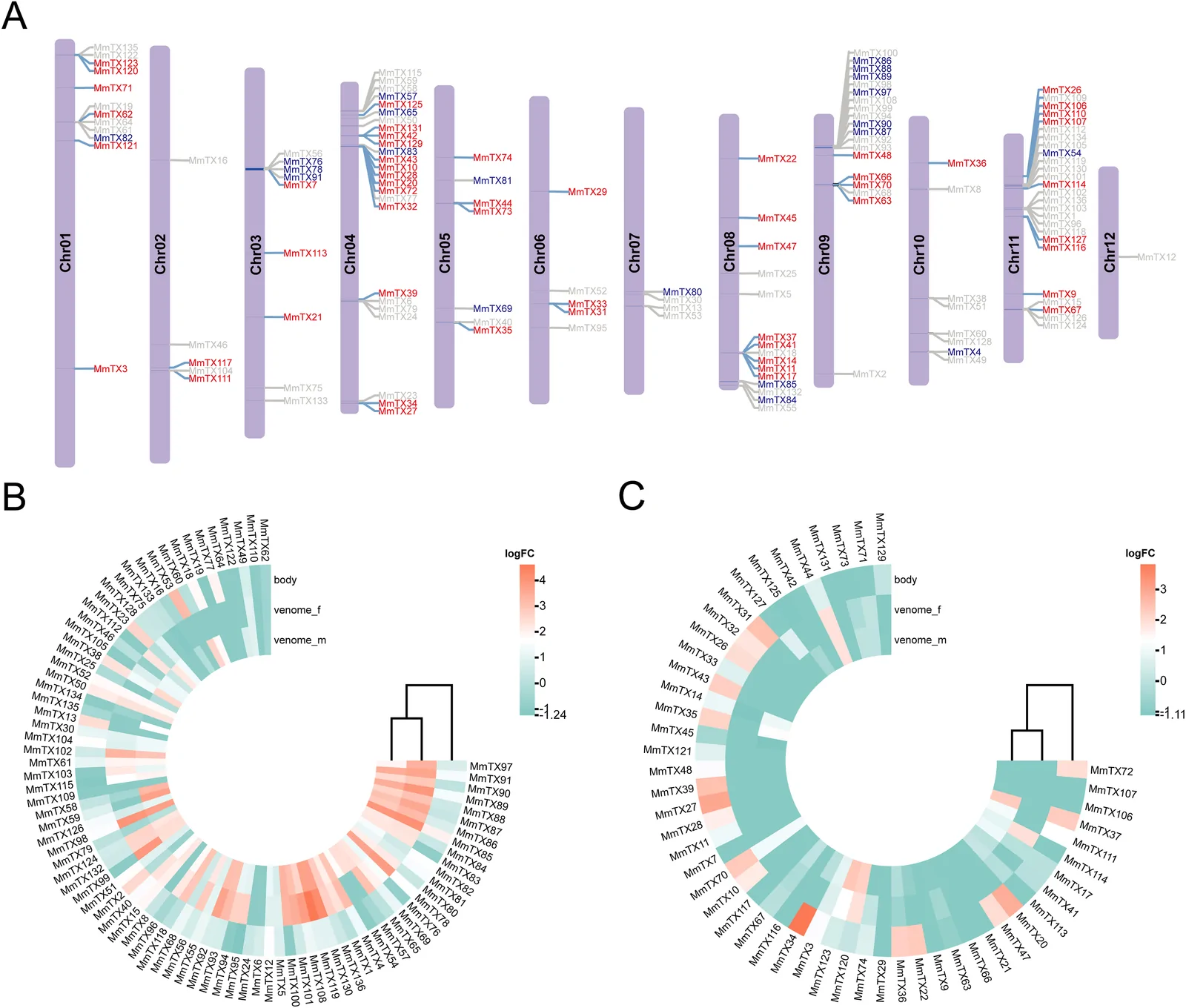
The diversity of venom peptides in *M. martensii*. (A) The venom peptides and their distribution on chromosomes. Blue: previously reviewed peptides, red: putative novel venom peptide candidates. (B) The expression of venom peptides. (C) The expression of 51 putative novel venom peptide candidates. (For interpretation of the references to colour in this figure legend, the reader is referred to the Web version of this article.)

### 3.4 Antithrombotic activity of venom peptides

To further validate the functional roles of venom peptides, we selected five Lys/Arg-rich peptides (MmTX67, MmTX70, MmTX106, MmTX118, and MmTX126) for heterologous expression and physiological activity characterization (Table S2). These peptides were prioritized using a combined strategy that considered Lys/Arg content, transcript abundance, sequence novelty, predicted peptide family, cysteine framework, and feasibility of recombinant expression. The five selected peptides were not exclusively chosen from the 51 putative novel venom peptide candidates; rather, they were selected from the broader venom peptide set to balance novelty, sequence features, and experimental tractability. Their high Lys/Arg content may favor electrostatic interactions with negatively charged catalytic pockets or membrane-associated coagulation reaction surfaces. The Lys/Arg contents of MmTX67, MmTX70, MmTX106, MmTX118, and MmTX126 were 21.43%, 15.79%, 15.15%, 20.00%, and 11.25%, respectively. These peptides range in length from 38 to 80 amino acids (AA) and contain 6 to 8 cysteine residues (Fig. 4A). MmTX67 consists of 42 AAs with 8 cysteine residues and is annotated as a potassium channel toxin. MmTX67 shares 52.5% sequence similarity with CeErgTx1 from *Centruroides elegans*. MmTX70, a 38 AA peptide with 6 cysteine residues, is also classified as a potassium channel toxin, displaying 51.43% sequence similarity to pMeKTx21-1 from *Mesobuthus eupeus*. MmTX106 contains 66 AAs eight of which are cysteine residues, and is categorized as a sodium channel toxin, exhibiting 68.18% sequence homology with BmKAEP2 from *M. martensii*. Similarly, MmTX118, another sodium channel toxin with 65 AAs and 8 cysteine residues, shows 96.92% sequence similarity to pMeNaTx11-2 from *Mesobuthus eupeus*. MmTX126, the longest among the selected peptides, comprises 80 AAs and 8 cysteine residues. It is also annotated as a sodium channel toxin, sharing 88.46% sequence identity with meuNaTx-4 from *Mesobuthus eupeus*. All venom peptides were heterologously expressed in *E. coli SHuffle* T7 using the pET-32a vector. Following purification, the peptides were rigorously validated through Tricine-SDS-PAGE to confirm their structural integrity and purity (Fig. S1).

**Fig. 4:**
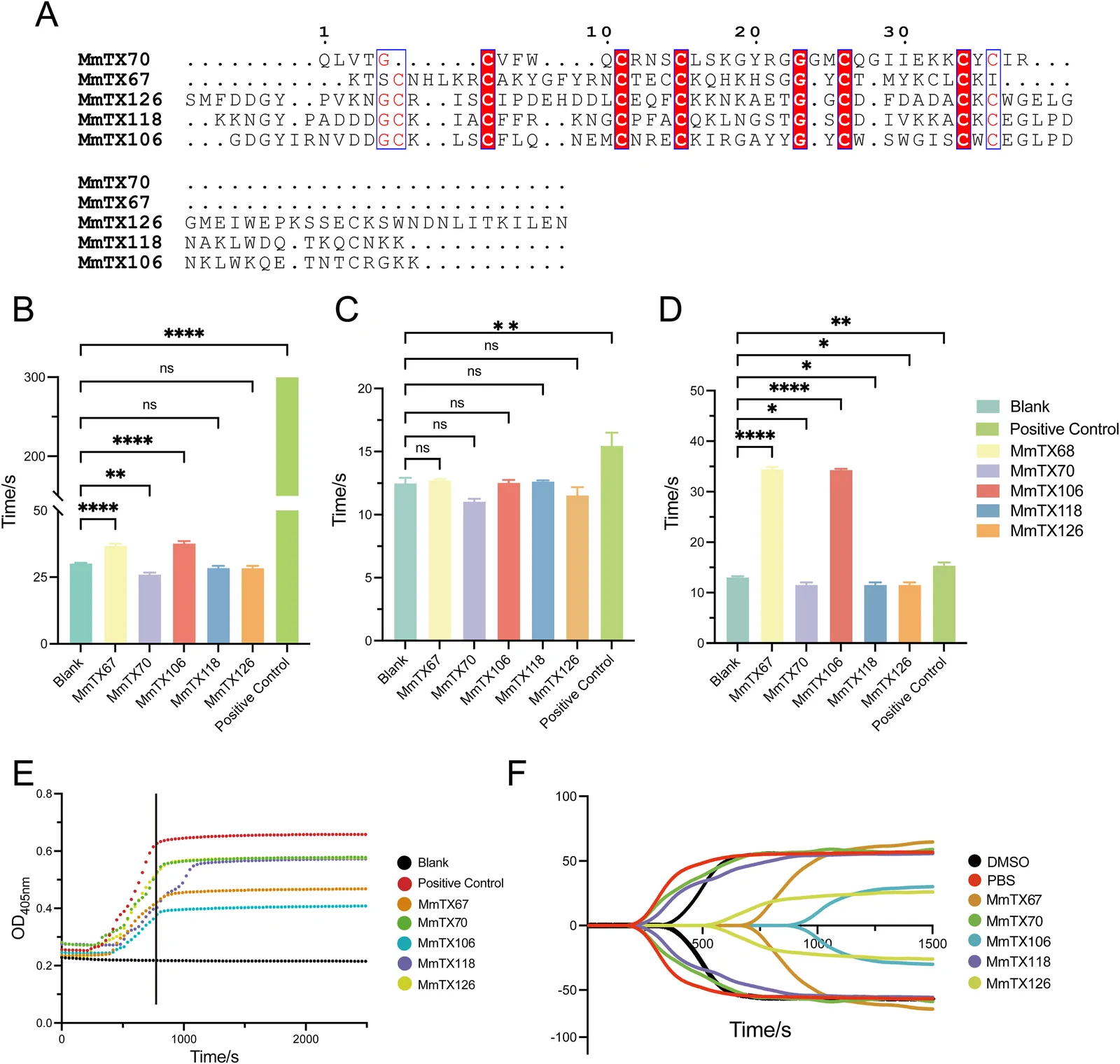
The antithrombotic activity of venom peptides in vitro. (A) The alignment of MmTX67, MmTX70, MmTX106, MmTX118 and MmTX126. APTT (B), PT (C), TT (D), recalcification assays (E) and TEG (F) analysis of MmTX67, MmTX70, MmTX106, MmTX118 and MmTX126. Columns show the mean values and standard deviation (mean ± SD, n = 3), and ****P < 0.0001, ***P < 0.001, **P < 0.01, *P < 0.05, ns, not significant (one-way ANOVA).

Systematic evaluation of the anticoagulant potential revealed distinct profiles among the five venom peptides. At 50 μg/mL, MmTX67, MmTX70, and MmTX106 demonstrated significant prolongation of APTT (Fig. 4B), while PT remained unaffected across all tested compounds (Fig. 4C). MmTX67 and MmTX106 demonstrated statistically significant prolongation of TT, extending the clotting duration by 2–3-fold at 50 μg/mL, thereby validating their potent interference with the final stage of fibrin formation in the coagulation cascade (Fig. 4D). Recalcification assays confirmed delayed fibrin formation after treatment with the five venom peptides (Fig. 4E). TEG analysis identified MmTX67, MmTX106, and MmTX126 as modulators of clotting kinetics, extending clot formation time (Fig. 4F). Collectively, these findings indicate that MmTX67 and MmTX106 exhibit relatively stronger *in vitro* anticoagulant activity among the tested peptides.

The *in vivo* antithrombotic effects of the venom peptides MmTX67, MmTX106, and MmTX126 were evaluated using a FeCl_3_-induced carotid artery thrombosis model in mice. The primary endpoints were the time to complete vascular occlusion and the area under the blood flow curve. The area under the curve (AUC) for blood flow was larger in all treatment groups compared to the model control group (Fig. 5A and S2-5). The AUC for the model group was 742,180 ± 390,012.15 (PU×s), while the MmTX67, MmTX106, and MmTX126 groups exhibited AUCs of 1,218,947.8 ± 474,969.20 (PU×s), 1,470,348.4 ± 374,352.53 (PU×s), and 1,365,704.8 ± 412,161.23 (PU×s), respectively. One-way ANOVA revealed a statistically significant difference only for the MmTX106 group compared to the model group, indicating a significant anticoagulant effect. Consistent with the AUC data, all treatment groups showed prolonged occlusion times (Fig. 5B). The mean occlusion time for the model group was 494 ± 134.28 s. In comparison, the occlusion times for the MmTX67, MmTX106, and MmTX126 groups were extended to 1001.2 ± 503.82 s, 1217.6 ± 292.78 s, and 1094.8 ± 549.87 s, respectively. Statistical analysis demonstrated that MmTX106 significantly prolonged the occlusion time compared to the model group. The antithrombotic effect was further corroborated by laser speckle blood flow imaging (Fig. 5C). At 8 min post-FeCl_3_ application, the blood flow signal (red area) in the model group had nearly disappeared, indicating complete occlusion. Conversely, the peptide-treated groups maintained better blood flow, with no complete occlusion observed. These data indicate that MmTX106 shows the most pronounced and statistically significant *in vivo* antithrombotic effect under the tested conditions, while MmTX67 and MmTX126 showed non-significant trends that require further validation.

**Fig. 5:**
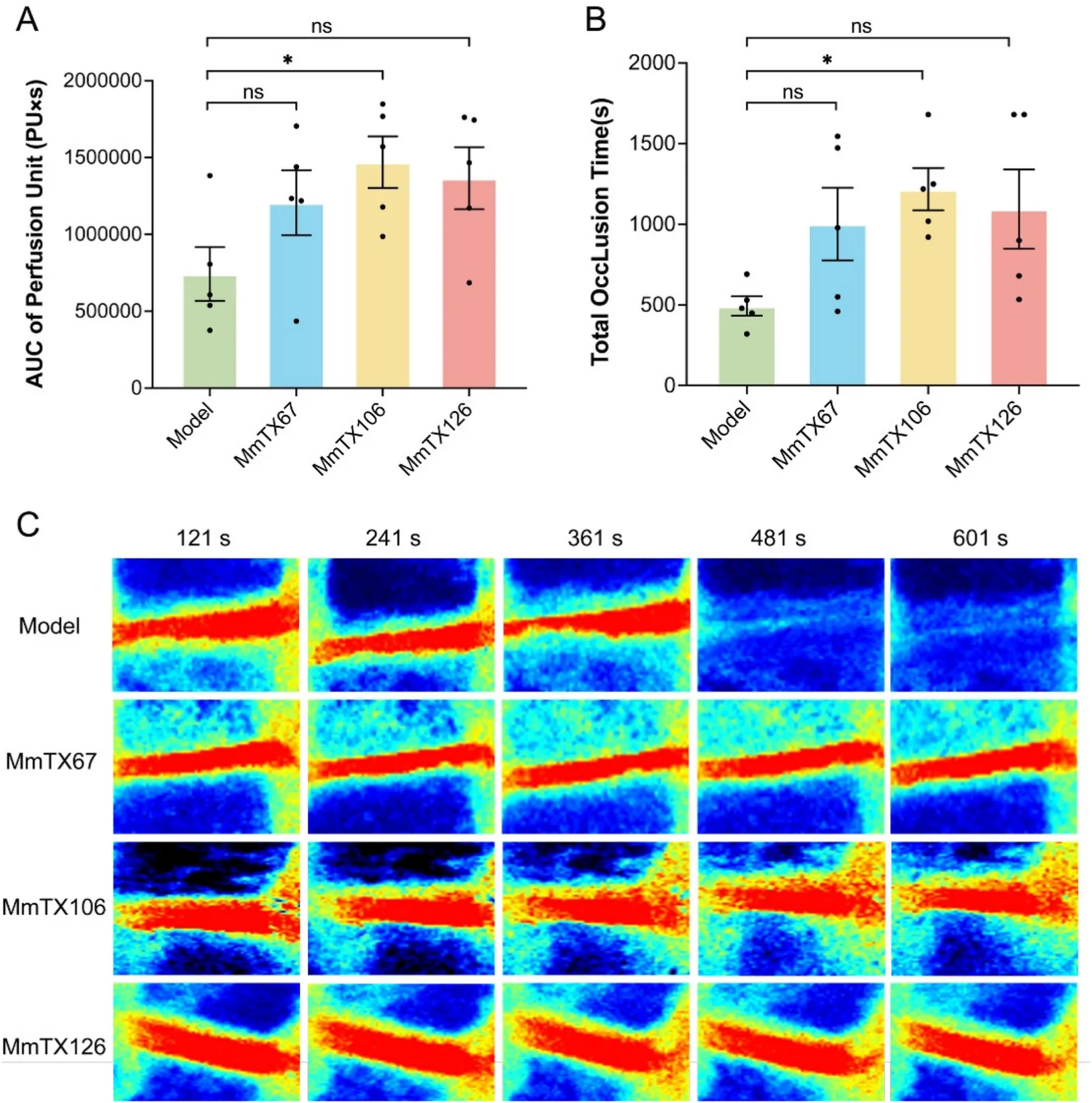
*In vivo* antithrombotic effects of venom peptides MmTX67, MmTX106, and MmTX126 in the FeCl_3_-induced carotid artery thrombosis model in mice. (A) Quantitative analysis of the area under the blood flow curve (AUC). The AUC values for the MmTX67, MmTX106 and MmTX126 treatment groups were all larger than that of the model control group. Statistical significance was observed only for the MmTX106 group compared to the model group (*P = 0.0395, one-way ANOVA). (B) Time to complete vascular occlusion. The mean occlusion time was prolonged in all treatment groups compared to the model group, with statistical significance achieved for MmTX106 (*P = 0.0321, one-way ANOVA). (C) Representative laser speckle blood flow images captured at 8 min post-FeCl_3_ application. The model group showed near-complete loss of blood flow signal (red area), indicating total vascular occlusion, whereas all peptide-treated groups maintained detectable blood flow without complete occlusion. Data are presented as mean ± SD (n = 5 mice per group). Statistical significance was determined by one-way ANOVA; *P < 0.05. (For interpretation of the references to colour in this figure legend, the reader is referred to the Web version of this article.)

## 4 Discussion

*M. martensii*, the source species of the traditional animal-derived medicine Quanxie, has long been used in China for conditions such as convulsions, rheumatic pain, and cerebrovascular disorders. However, the active material basis of Quanxie has largely remained at the level of crude extracts or complex mixtures, with limited systematic molecular resolution. To elucidate the genetic architecture underlying scorpion-derived pharmacologically active compounds, this study achieved a chromosome-level genome assembly for *M. martensii*. The resulting genome provides an excellent scaffold for investigating genomic architecture, gene localization, and evolutionary dynamics in this species, establishing a resource for future functional and comparative genomic studies. The genome assembly of *M. martensii*, generated using Hifiasm, spanned 1.08 Gb with a contig N50 of 46.46 Mb (Fig. 1). This represents a substantial improvement over the 2013 reference genome (assembly size: 925.5 Mb; contig N50: 45.2 kb) [24]. Compared with approaches that mainly rely on venom-gland transcriptomes, a genome-guided resource can more accurately resolve the organization, expansion, and evolutionary relationships of venom peptide genes, and can reduce omissions caused by tissue-specific or low-level expression. This genome therefore provides an important reference not only for studying the active material basis of Quanxie, but also for comparative and evolutionary analyses of scorpion venom systems. While this assembly provides a robust foundation, annotation accuracy remains dependent on existing genomic databases and computational tools. Future efforts should integrate long-read transcriptomic and proteomic datasets to refine structural and functional annotations [30], further enhancing the genome's resolution for evolutionary and mechanistic investigations.

Using an integrated genomic and transcriptomic strategy, this study identified 51 novel venom peptide candidates, supporting multi-omics analysis as an effective route for discovering natural bioactive peptides. Traditional venom peptide discovery has depended heavily on venom extraction and proteomic analysis [14,15,31,32], but these approaches can be constrained by expression abundance, sample source, and analytical sensitivity. As a result, low-abundance peptides or components expressed under specific physiological conditions may be missed. By combining genome-based prediction with transcriptional evidence, this study improves the accuracy and completeness of venom peptide identification and expands the known peptide repertoire of *M. martensii.* These newly identified candidates enrich the structural diversity of scorpion venom peptides and provide a resource for subsequent pharmacological screening and structure-function studies.

The selection of the five candidates was guided by their enrichment in positively charged residues, particularly lysine and arginine. Their positively charged side chains mediate electrostatic interactions with negatively charged regions, which is relevant to coagulation serine proteases. Natural inhibitors often use arginine or lysine as the P1 residue, forming ionic bonds with the Asp189-centered negatively charged S1 pocket of target enzymes, such as thrombin and factor Xa. Additionally, negatively charged phospholipid membranes are crucial for coagulation complex assembly and function [33,34]. Therefore, these Lys/Arg-rich venom peptides represent rational antithrombotic candidates via active-site or surface modulation. Further functional screening showed that MmTX67, MmTX106, and MmTX126 modulated clotting parameters *in vitro*, and MmTX106 further showed a favorable antithrombotic effect *in vivo*. These findings provide a potential modern pharmacological clue for the traditional use of *Quanxie* in diseases associated with stroke or blood stasis. Thrombotic disorders remain a leading cause of morbidity and mortality worldwide, with conditions such as acute myocardial infarction, ischemic stroke, and venous thromboembolism posing substantial clinical and socioeconomic burdens [[35], [36], [37]]. Despite the availability of anticoagulants and antiplatelet agents, current pharmacotherapy is hampered by significant limitations, including narrow therapeutic windows, unpredictable drug responses, and, most critically, an inherent risk of major bleeding complications [38]. Venom-derived peptides have become important sources for drug discovery because of their potency, target selectivity, and distinctive structural features. Several snake-venom-derived molecules have already entered clinical use, whereas scorpion venom peptides remain comparatively underexplored in the antithrombotic field [[39], [40], [41], [42], [43]]. The activity profile of MmTX106 therefore broadens the known pharmacological spectrum of scorpion venom peptides and suggests that *M. martensii* may contain lead molecules for hemostasis-related drug discovery. Although their direct molecular targets remain unresolved, the peptides did not inhibit platelet aggregation,suggesting that inhibition of platelet aggregation is unlikely to be the primary mechanism under the conditions tested. Their coagulation profiles, particularly prolonged APTT with unchanged PT, indicate preferential modulation of the intrinsic coagulation pathway [44,45]. The active peptides may therefore target intrinsic coagulation factors or their regulatory interactions. Further studies should identify their direct targets and clarify their effects on coagulation kinetics. Structural and computational analyses may then support structure–activity studies and peptide optimization [17,46].

## 5 Limitations

However, several limitations should be considered. First, from the broader venom peptide set, only five candidates were recombinantly expressed and functionally validated, leaving the biological activities of many peptides unexplored. Second, the current pharmacological evaluation focused mainly on antithrombotic activity, whereas other possible activities, such as anti-inflammatory, analgesic, and antitumor effects, were not systematically assessed. Third, although MmTX106 showed promising in vivo activity and its coagulation assay profile suggests potential modulation of coagulation-related processes, its direct molecular targets remain to be identified. In particular, whether MmTX106 acts on specific coagulation factors, regulatory protein-protein interactions, or membrane-associated coagulation complexes requires further investigation. Finally, key translational issues, including pharmacokinetics, safety, immunogenicity, long-term toxicity, and bleeding risk, still require further evaluation before the clinical potential of MmTX106 can be assessed.In addition, because only male mice were included, it remains unknown whether the observed effects are sex-dependent.

## 6 Conclusion

In summary, this study constructed a high-quality reference genome for *M. martensii,* systematically identified a set of novel venom peptide resources through multi-omics analysis, and identified MmTX106 as the most promising antithrombotic candidate, showing significant activity both *in vitro* and *in vivo*, whereas MmTX67 and MmTX126 displayed non-significant *in vivo* trends that warrant further validation. These findings deepen our understanding of the genetic basis of active substances in *Quanxie* and provide molecular evidence for interpreting the modern scientific basis of this traditional animal-derived medicine. More broadly, the venom peptide discovery and functional evaluation framework established here offers a useful paradigm for developing animal venom-derived peptides and provides lead candidates for future antithrombotic studies.

## CRediT authorship contribution statement

**Daixi Liu:** Writing – review & editing, Writing – original draft, Project administration, Funding acquisition, Data curation, Conceptualization. **Qianhui Zeng:** Project administration, Methodology. **Yifei Sun:** Validation. **Changfen Li:** Validation. **Yanjun Song:** Writing – review & editing. **Zhuo Shang:** Writing – review & editing. **Kunlong Li:** Writing – review & editing. **Hongjie Zhu:** Writing – review & editing. **Feng Li:** Writing – review & editing. **Chuanbin Shen:** Writing – review & editing, Validation, Data curation. **Jianhua Ju:** Writing – review & editing, Supervision, Project administration, Funding acquisition.

## Data availability

All raw sequencing data and genome assemblies are archived at the Genome Sequence Archive of China National Center for Bioinformation (https://www.cncb.ac.cn/) under project PRJCA038539.

## Ethics approval

All animal experiments were approved by the Institutional Animal Care and Use Committee of Shandong University (approval No. YXDW2025-0099) and were conducted in accordance with institutional animal care guidelines and ARRIVE reporting principles.

## Declaration of generative AI in scientific writing

No generative AI tools have been used throughout the entire writing process of this manuscript.

## Funding information

This work was supported by the 10.13039/501100007129Shandong Provincial Natural Science Foundation Major Basic Research Project (ZR2024ZD42), the 10.13039/501100001809National Natural Science Foundation of China (21310005202508), and the Shandong Laboratory Program (SYS202205).

## Declaration of interests

The authors declare the following financial interests/personal relationships which may be considered as potential competing interests:

Daixi Liu, Qianhui Zeng, and Jianhua Ju are inventors on patent ZL202510864842.X, which is assigned to Shandong University. In addition, the author of this manuscript, Jianhua Ju, serves as an Executive Editor of this journal; however, he was not involved in the peer review or editorial decision-making for this manuscript.The authors declare no other competing financial interests or personal relationships that could have appeared to influence the work reported in this paper.

## References

[1] Cao Z., Di Z., Wu Y., Li W. (2014). Overview of scorpion species from China and their toxins. Toxins.

[2] Wong K.L., Wong R.N.S., Zhang L., Liu W.K., Ng T.B., Shaw P.C. (2014). Bioactive proteins and peptides isolated from Chinese medicines with pharmaceutical potential. Chin. Med..

[3] Li Z., Hu P., Wu W., Wang Y. (2019). Peptides with therapeutic potential in the venom of the scorpion *Buthus martensii* Karsch. Peptides.

[4] Ortiz E., Gurrola G.B., Schwartz E.F., Possani L.D. (2015). Scorpion venom components as potential candidates for drug development. Toxicon.

[5] Uzair B., Bint-E-Irshad S., Khan B.A., Azad B., Mahmood T. (2018). Scorpion venom peptides as a potential source for human drug candidates. Protein Pept. Lett..

[6] Xia Z., He D., Wu Y., Kwok H.F., Cao Z. (2023). Scorpion venom peptides: molecular diversity, structural characteristics, and therapeutic use from channelopathies to viral infections and cancers. Pharmacol. Res..

[7] Guo Y., Zhu W., Yuan P., Huang X., Lu S., Cao Z. (2024). Similar neurotoxin expression profiles of traditional Chinese scorpion medicine material between juvenile and adult *Mesobuthus martensii* scorpions revealed by multiple strategic proteomics. J. Ethnopharmacol..

[8] Xin K., Sun R., Xiao W., Lu W., Sun C., Lou J. (2025). Short peptides from Asian scorpions: bioactive molecules with promising therapeutic potential. Toxins.

[9] Ji Y., Li Y., Zhang J., Song B., Yamaki T., Mochizuki T. (1999). Covalent structures of BmK AS and BmK AS-1, two novel bioactive polypeptides purified from Chinese scorpion *Buthus martensi* Karsch. Toxicon.

[10] Bai Z., Liu T., Pang X., Chai Z., Ji Y. (2007). Suppression by intrathecal BmK IT2 on rat spontaneous pain behaviors and spinal c-Fos expression induced by formalin. Brain Res. Bull..

[11] de Melo M.M.A., Oliveira V. da S., de Queiroz Neto M.F., Paiva W. de S., Torres-Rêgo M., Silva S.R.B. (2022). TanP: a multifunctional anionic peptide from *Tityus stigmurus* scorpion venom. Front. Mol. Biosci..

[12] Tran T.V., Hoang A.N., Nguyen T.T.T., Phung T.V., Nguyen K.C., Osipov A.V. (2017). Anticoagulant activity of low-molecular weight compounds from *Heterometrus laoticus* scorpion venom. Toxins.

[13] Lee A., Cole B.L., Ojemann J., Kittle D., Perry J., Parrish-Novak J. (2025). Phase 1 and expanded imaging study of tozuleristide in patients with pediatric primary central nervous system tumors. J. Neurosurg. Pediatr..

[14] So W.L., Leung T.C.N., Nong W., Bendena W.G., Ngai S.M., Hui J.H.L. (2021). Transcriptomic and proteomic analyses of venom glands from scorpions *Liocheles australasiae, Mesobuthus martensii*, and *Scorpio maurus palmatus*. Peptides.

[15] Xu X., Duan Z., Di Z., He Y., Li J., Li Z. (2014). Proteomic analysis of the venom from the scorpion *Mesobuthus martensii*. J. Proteomics.

[16] Smallwood T.B., Clark R.J. (2021). Advances in venom peptide drug discovery: where are we at and where are we heading?. Expert Opin. Drug Discov..

[17] Muttenthaler M., King G.F., Adams D.J., Alewood P.F. (2021). Trends in peptide drug discovery. Nat. Rev. Drug Discov..

[18] Lu B., Jiang J., Wu H., Chen X., Song X., Liao W. (2021). A large genome with chromosome-scale assembly sheds light on the evolutionary success of a true toad (*Bufo gargarizans*). Mol. Ecol. Resour..

[19] Guang X., Lan T., Wan Q., Huang Y., Li H., Zhang M. (2021). Chromosome-scale genomes provide new insights into subspecies divergence and evolutionary characteristics of the giant panda. Sci. Bull..

[20] Zheng Y., Yang D., Yin X., Yang X., Chen M., Li X. (2024). The chromosome-level genome assembly of *Cananga odorata* provides insights into its evolution and terpenoid biosynthesis. New Phytol..

[21] Bryant M.J., Coello A.M., Glendening A.M., Hilliman S.A., Jara C.F. (2024). Unveiling the genetic blueprint of a desert scorpion: a chromosome-level genome of *Hadrurus arizonensis* provides the first reference for Parvorder Iurida. Genome Biol. Evol..

[22] Su Y., Xiu M., Yang H., Shi C. (2025). A chromosome-level genome assembly for the desert scorpion *Mesobuthus przewalskii* from Asian drylands. J. Hered..

[23] Guirao-Rico S., Pisarenco V.A., Escuer P., Balart-García P., Domènech M. (2026). ERGA-CBP chromosome-level genome assembly of the blind scorpion *Belisarius xambeui* Simon, 1879 (Belisariidae, Scorpiones), a singular scorpion in Europe. Open Res. Eur..

[24] Cao Z., Yu Y., Wu Y., Hao P., Di Z., He Y. (2013). The genome of *Mesobuthus martensii* reveals a unique adaptation model of arthropods. Nat. Commun..

[25] Yamashita T., Rhoads D.D., Pummill J. (2024). A robust genome assembly with transcriptomic data from the striped bark scorpion. Centruroides vittatus, G3 (Bethesda).

[26] El Ghoubali D., Pirro S., Sehli S., Merzouki M., Idrissi N.A., Belyamani L. (2022). The complete genome sequence of *Androctonus mauritanicus*, the Moroccan black thick-tailed scorpion. Biodivers. Genomes.

[27] Scarano C., Veneruso I., De Simone R.R., Di Bonito G., Secondino A., D'Argenio V. (2024). The third-generation sequencing challenge: novel insights for the omic sciences. Biomolecules.

[28] Chen C., Wu Y., Li J., Wang X., Zeng Z., Xu J. (2023). TBtools-II: a “one for all, all for one” bioinformatics platform for biological big-data mining. Mol. Plant.

[29] Chen C., Chen H., Zhang Y., Thomas H.R., Frank M.H., He Y. (2020). TBtools: an integrative toolkit developed for interactive analyses of big biological data. Mol. Plant.

[30] Monzó C., Liu T., Conesa A. (2025). Transcriptomics in the era of long-read sequencing. Nat. Rev. Genet..

[31] Gao S., Liang H., Shou Z., Yao Y., Lv Y., Shang J. (2021). De novo transcriptomic and proteomic analysis and potential toxin screening of *Mesobuthus martensii* samples from four different provinces. J. Ethnopharmacol..

[32] Grashof D.G.B., Kerkkamp H.M.I., Afonso S., Archer J., Harris D.J., Richardson M.K. (2019). Transcriptome annotation and characterization of novel toxins in six scorpion species. BMC Genom..

[33] Otlewski J., Jelen F., Zakrzewska M., Oleksy A. (2005). The many faces of protease-protein inhibitor interaction. EMBO J..

[34] Üstok F.I., Faille A., Huntington J.A. (2026). A 3.3-Å cryo-EM structure of an engineered high-affinity human prothrombinase complex. Blood.

[35] Lindstrom M., DeCleene N., Dorsey H., Fuster V., Johnson C.O., LeGrand K.E. (2022). Global burden of cardiovascular diseases and risks collaboration, 1990–2021. J. Am. Coll. Cardiol..

[36] Fredenburgh J.C., Weitz J.I. (2021). Factor XI as a target for new anticoagulants. Hämostaseologie.

[37] Weitz J.I., Fredenburgh J.C. (2017). Factors XI and XII as targets for new anticoagulants. Front. Med..

[38] Zhang W., Pei B., Zhou Y., Li H., Ma W., Zhou B. (2026). Emerging targets, novel directions, and innovative approaches in thrombosis therapy. Aging Dis..

[39] Koh C.Y., Kini R.M. (2012). From snake venom toxins to therapeutics–cardiovascular examples. Toxicon.

[40] Lazarovici P., Marcinkiewicz C., Lelkes P.I. (2019). From snake venom's disintegrins and C-type lectins to anti-platelet drugs. Toxins.

[41] Slagboom J., Mladić M., Xie C., Kazandjian T.D., Vonk F., Somsen G.W. (2020). High throughput screening and identification of coagulopathic snake venom proteins and peptides using nanofractionation and proteomics approaches. PLoS Negl. Trop. Dis..

[42] Mohamed Abd El-Aziz T., Soares A.G., Stockand J.D. (2019). Snake venoms in drug discovery: valuable therapeutic tools for life saving. Toxins.

[43] Debono J., Dobson J., Casewell N.R., Romilio A., Li B., Kurniawan N. (2017). Coagulating colubrids: evolutionary, pathophysiological and biodiscovery implications of venom variations between boomslang (*Dispholidus typus*) and twig snake (*Thelotornis mossambicanus*). Toxins.

[44] Mackman N. (2008). Triggers, targets and treatments for thrombosis. Nature.

[45] He M., He Y., Feng X., Li Z., Lin T., Yang J. (2026). Bannakunin: a dual-target Kunitz inhibitor bridging anticoagulation (FXa/XIIa) and anti-platelet (α2β1/P2Y12) pathways. Cell. Mol. Biol. Lett..

[46] Otvos L. (2024). The latest trends in peptide drug discovery and future challenges. Expert Opin. Drug Discov..

