# Supplementary material for: The chromosome-level genome of Mesobuthus martensii provides insights into its evolution and the diversity of venom peptides
Source: Pharm Sci Adv. 2026 Sep 6;4:100149. doi: 10.1016/j.pscia.2026.100149 (PMC13679368; doi:10.1016/j.pscia.2026.100149)
Supplement: Supplementary file 1 [file mmc1.docx]

**The chromosome-level genome of *Mesobuthus martensii* provides insights into its evolution and the diversity of venom peptides**

Daixi Liu ^a,1^^,*^, Qianhui Zeng ^a,1^, Yifei Sun ^a^,Changfen Li ^a^, Yanjun Song ^a^, Zhuo Shang ^a^, Kunlong Li ^a^, Hongjie Zhu ^a^, Feng Li ^a^, Chuanbin Shen ^b,*^, Jianhua Ju ^a^^,*^

^a^ State Key Laboratory of Discovery and Utilization of Functional Components in Traditional Chinese Medicine, Key Laboratory of Chemical Biology (Ministry of Education), Shandong Basic Science Research Center (Pharmacy), School of Pharmaceutical Sciences, Cheeloo College of Medicine, Shandong University, Jinan 250012, China.

^b^ Key Laboratory of Marine Drugs, Chinese Ministry of Education, School of Medicine and Pharmacy, Ocean University of China, Qingdao 266000, China

*Corresponding authors.

E-mail addresses: [liudaixi@sdu.edu.cn](mailto:liudaixi@sdu.edu.cn) (Daixi Liu), [shenchuanbin@ouc.edu.cn](mailto:shenchuanbin@ouc.edu.cn) (Chuanbin Shen), [jju@sdu.edu.cn](mailto:jju@sdu.edu.cn) (Jianhua Ju).

^1^ These authors contributed equally to this work.

**Supporting Information**

**List of Supplemental Tables**

Table S1. Comparison of different versions of *M. martensii* genome

Table S2. *M. martensii* venom peptides selected for functional characterization*.*

Table S3. List of 136 venom peptides identified via HMM-based homology search.

Table S4. Primer sequences used in this study.

**List of Supplemental Figures**

Figure S1. *M. martensii.*

Figure S2. Tricine-SDS-PAGE of scorpion venom peptides.

Figure S3. Blood flow imaging status in the model group of mouse common carotid artery thrombosis test.

Figure S4. Blood flow imaging status in the mmtx67 group of mouse common carotid artery thrombosis test.

Figure S5. Blood flow imaging status in the mmtx106 group of mouse common carotid artery thrombosis test.

Figure S6. Blood flow imaging status in the mmtx126 group of mouse common carotid artery thrombosis test.

**Table S1. Comparison of different versions of *M. martensii* genome**

| Genome Version | Version 2013 | Version 2025 |
| --- | --- | --- |
| Assemble | Contig | Chromosome |
| Contig N50 | 45.2 Kb | 46.46 Mb |
| BUSCO | 57.13% | 97.13% |
| Protein-encoding genes | 32,016 | 26,533 |

**Table S2. *M. martensii* venom peptides selected for functional characterization*.***

| **No.** | **AA** | **Cys** | **Proposed function** | **Identities（%）** | **Protein homolog/origin** | **Lys + Arg (%)** | **Transcript abundance** |
| --- | --- | --- | --- | --- | --- | --- | --- |
| MmTX67 | 42 | 8 | potassium channel toxin | 52.5 | CeErgTx1(Q86QV6.1), *Centruroides elegans* | 21.43% | 1.213157 |
| MmTX70 | 38 | 6 | potassium channel toxin | 51.43 | pMeKTx21-1(AIL48797.1), *Mesobuthus eupeus* | 15.79% | 12.49533 |
| MmTX106 | 66 | 8 | sodium channel toxin | 68.18 | BmKAEP2(Q86M31.1), *Olivierus martensii* | 15.15% | 0 |
| MmTX118 | 65 | 8 | sodium channel toxin | 96.92 | pMeNaTx11-2(XHB13757.1), *Mesobuthus eupeus* | 20.00% | 935.430908 |
| MmTX126 | 80 | 8 | sodium channel toxin | 88.46 | meuNaTx-4 ([AMX81462.1](https://www.ncbi.nlm.nih.gov/protein/AMX81462.1?report=genbank&log$=protalign&blast_rank=1&RID=W24JFT8T016)), *Mesobuthus eupeus* | 11.25% | 12.474041 |

**Table S4. Primer sequences used in this study.**

| Primer | Sequence |
| --- | --- |
| 32a-MmTX67-F | GACGACGACGACAAGAAAACTAGCTGCAATCATTTAAAGC |
| 32a-MmTX67-R | GTGGTGGTGGTGGTGGTGTTATATTTTACATAGGCATTTATACATTGTA |
| 32a-MmTX70-F | GACGACGACGACAAGTCAATGTTCGATGATGGATATCCAG |
| 32a-MmTX70-R | CAGTGGTGGTGGTGGTGGTGTTAATTTTCCAAAATCTTTGTTATCAGATTGTC |
| 32a-MmTX106-F | GACGACGACGACAAGGGTGATGGTTACATAAGAAATGTAGA |
| 32a-MmTX106-R | GTGGTGGTGGTGGTGGTGTTACTTTTTCCCTCTACATGTATTGGT |
| 32a-MmTX118-F | GACGACGACGACAAGAAAAAGAATGGTTATCCAGCAGAT |
| 32a-MmTX118-R | CAGTGGTGGTGGTGGTGGTGTTATTTTTTGTTGCATTGTTTGGTTTGAT |
| 32a-MmTX126-F | GACGACGACGACAAGTCAATGTTCGATGATGGATATCCAG |
| 32a-MmTX126-R | CAGTGGTGGTGGTGGTGGTGTTAATTTTCCAAAATCTTTGTTATTAGATTGTCAT |

**Figure S1. *M. martensii.***

**Figure S2. Tricine-SDS-PAGE of scorpion venom peptides.**

**Figure S3. Blood flow imaging status in the model group of mouse common carotid artery thrombosis test.**

**Figure S4. Blood flow imaging status in the MmTX67 group of mouse common carotid artery thrombosis test.**

**Figure S5. Blood flow imaging status in the MmTX106 group of mouse common carotid artery thrombosis test.**

**Figure S6. Blood flow imaging status in the MmTX126 group of mouse common carotid artery thrombosis test.**
